# Downregulated NM23-H1 expression is associated with intracranial invasion of nasopharyngeal carcinoma

**DOI:** 10.1038/sj.bjc.6604167

**Published:** 2008-01-22

**Authors:** S J Liu, Y M Sun, D F Tian, Y C He, L Zeng, Y He, C Q Ling, S H Sun

**Affiliations:** 1 Faculty of Integrative Medicine, Traditional Chinese Medicine University of Hunan, Changsha, People's Republic of China; 2 Department of Traditional Chinese Medicine, Changhai Hospital, Shanghai, People's Republic of China; 3 Institute of Genetics and Developmental Biology, Chinese Academy of Sciences, Beijing, People's Republic of China; 4 Department of Pathology, Hunan Provincial Tumor Hospital, Changsha, People's Republic of China; 5 Department of Medical Genetics, Second Military Medical University, Shanghai, People's Republic of China

**Keywords:** NPC, intracranial invasion, gene chip, NM23-H1, berberine

## Abstract

Because the focus of nasopharyngeal carcinoma (NPC) is very close to intracranial organs, it often makes incursions into cranial cavity. Identification of intracranial invasion-associated indicators will provide potential therapeutic targets for NPC patients with intracranial invasion. In this regard, Human Xpro™ HC-plus cancer-related gene chip was utilised to screen intracranial invasion-associated genes for NPC from the biopsied primary focus tissue samples. In all, 8 upregulated and 23 downregulated genes were obtained. VEGF165 and MMP-9, the two upregulated genes, and NM23-H1, the downregulated one, were further confirmed by immunohistochemistry, quantitative real-time PCR and western blot. Invasion-associated cellular and nude mouse models were subsequently employed to study the biological properties of NM23-H1. NM23-H1 expression was significantly lower in 5-8F cells compared with that in 6-10B cells. Moreover, patch-clamp and transwell chamber were adopted to investigate the invading potential-associated biological dynamic mechanisms in the two cell lines, and Ca^2+^ current and motility were significantly elevated in 5-8F cells compared with that in 6-10B cells. Berberine, an inhibitor of Ca^2+^ current, could substantially increase the expression of NM23-H1 and decrease 5-8F cell motility. The specificity of berberine on NM23-H1 and cell motility was confirmed by RNAi assay.

Nasopharyngeal carcinoma (NPC) is the most common epithelial malignant neoplasm in the nasopharynx, especially in Southern China. Because of its close location to the intracranial organs, cranial cavity invasion and metastasis that lead to poor prognosis are commonly observed in clinical practice. Therefore, a better understanding of molecular mechanisms involving in NPC intracranial invasion and metastasis may lead to more effective treatment of NPC patients.

Recently, gene-expression profiles provide more important information about cancerous molecular biology and were used to explore candidate biomarkers and identify groups of genes involved in tumorigenesis. Several gene-expressing profiling procedures have been applied to the various studies of NPC (Nie *et al*, 2003; Sung *et al*, 2007; Zeng *et al*, 2007). These studies have provided important information and led to the discovery of a number of genes useful for laboratory research for NPC detection, diagnosis and treatment. However, no gene-expressing profile studies have been involved in intracranial invasion, and metastasis-related genes in NPC tissue samples taken from the primary focus in the nasopharynx.

Nucleoside diphosphate kinases, a highly conserved family in eukaryotes, are encoded by NM23 genes. NM23-H1-8 has been identified in human (Lacombe *et al*, 1993). NM23-H1 catalyses the phosphorylation of nucleoside 5′-diphosphates to triphosphates through a ‘ping-pong’ mechanism involving a high-energy phosphorylated enzyme intermediate, thus equilibrating the cellular pool of NDP and NTP (Lascu and Gonin, 2000). NM23-H1 was identified by Steeg *et al* (1988a, 1988b), based on its low expression in metastatic cells. Since then, low expression or mutation of NM23-H1 has been implicated in cancer prognosis or metastasis in a variety of tumours and malignant transformation (Leone *et al*, 1991; Yih *et al*, 2002; Fan *et al*, 2003; Zhao and Li, 2004; Ma *et al*, 2005). However, it is still unclear whether NM23-H1 expression is decreased or not and whether it is associated with NPC intracranial Invasion.

In the present study, we used human cancer-relating gene chip to determine the differences in gene expression between NPCs from the primary nasopharyngeal focuses with and without intracranial invasion and identified a number of differentially expressed genes, including NM23-H1, that might be involved in the intracranial invasion of NPC. Then, we adopted invasion-associated cellular and nude mouse models to study the role of NM23-H1 in NPC cell migration and metastasis.

## MATERIALS AND METHODS

### Patients and tissue samples of tumour

The primary focus tissue samples of NPC were obtained from NPC patients in Hunan Provincial Tumor Hospital (Changsha, China), including tissue specimens taken from NPC patients with intracranial invasion (seven men and three women; age ranged from 20 to 63 years) and without invasion (seven men and three women; age ranged from 20 to 63 years). All the patients were first diagnosed in this hospital without treatment. They did not have a familial history of NPC. There were no significant statistic differences in the distribution of gender and age in this group of cases (Table 1). The cases with intracranial invasion from the lesion were diagnosed by magnetic resonance imaging (Ng *et al*, 1997). All patients underwent endoscopies for medical reasons and gave informed consent to take part in the study. This study was carried out with ethical committee approval. The tissue specimens from the primary focus (confirmed pathohistologically as squamous cell carcinoma) were snap frozen in liquid nitrogen and underwent blinded analysis (preparations for gene chip and gene chip results analysis).

### Gene-expression profiling

The differentially expressed genes related to NPC intracranial invasion or metastasis potential were screened with Human Xpro™ HC-plus cancer-relating gene chip of oligonucleotide (2748 cancer-relating genes; National Engineering Research Center for Miniaturized Detection System, Xian, China). The experimental procedures were carried out in a comparative way between groups of biopsies from the primary nasopharyngeal focus without invasion or metastasis and those with intracranial invasion. Total RNA was reverse transcribed with Cy3-labelled deoxyuridine triphosphate incorporation and fluorescence quantified using a Gene Pix 4000A Microarray Scanner (Gene Pix Pro 3.0 software; Axon Instruments Inc., Union, CA, USA). The gene-expressing profiling has been repeated three times for each group, and six chips have been performed for the 20 patients. Subsequent analysis was conducted by Spotfire 8.0 software.

### Animals, agents and antibody

Animal experiments were performed in accordance with the regulations of our institution's ethics commission and with the United Kingdom Co-ordinating Committee on Cancer Research guidelines (Workman *et al*, 1998). Six-week-old male BALB/c nude mice were purchased from the Institute of Laboratory Animal Science, Chinese Academy of Medical Science. Berberine was obtained from Sigma (St Louis, MO, USA). Mouse monoclonal antibody for NM23-H1 and MMP-9 were from Maxin, Fuzhou, China and rabbit polyclonal antibody for VEGF165 was from Santa Cruz, California, USA.

### Tumour cell lines and transfection

Human NPC cell lines 5-8F and 6-10B (established by Oncology Institute of Sun Yat-sen University Cancer Center) were grown in RPMI 1640 (Gibco, BRL, USA) supplemented with 10% fetal calf serum (Sigma). Cells were transfected with Lipofectamine™ 2000 (Invitrogen, California, USA) according to the manufacturer's instructions.

### siRNA experiment

Hairpin loop constructs that produce specific small-interfering RNAs (siRNAs; siNM23-H1 in text and figures) were designed and constructed by targeting GGAGAUCGGCUUGUGGUUU and GCUUCCGAAGAUCUUCUCA, two corresponding coding regions of human NM23-H1 and a nonspecific control siRNA GCGCGGGGCACGUUGGUGU (Yao *et al*, 2005). Annealed oligonucleotides were cloned into the *Bam*HI/*Eco*RI site of RNAi-Ready pSIREN-RetroQ-ZsGreen (BD Biosciences, Palo Alto, CA, USA). 5-8F cells (2 × 10^5^ cells per well) were plated the day before transfection.

### Quantitative real-time PCR

Total RNA was extracted from the tissue samples using the Quick Prep Total RNA Extraction Kit (Qiagen, Valencia, CA, USA). Total RNA (1 *μ*g) was reverse transcribed using Oligo (dT)_18_ Primer and SuperScript™ III (Invitrogen) to synthesise complementary DNA (cDNA) following standard protocols. Transcriptional level was quantified by quantitative real-time PCR using the Syber Green PCR MASTER MIX kit (Applied Biosystems, Foster City, CA, USA). Amplification and detection were performed with ABI PRISM 7300 Sequence Detection System starting with 1 *μ*l of cDNA. The following primers and probes were used NM23-H1, CCGGAGTTCAAACCTAAGCA and AGTTCCTCAGGGTGAAACCA; VEGF165: ACTTTCTGCTGTCTTGGGTG and CATCTGCAAGTACGTTCGTTT; and MMP-9: ACCTTCACTCGCGTGTACAGC and GCGGAGTAGGATTGGCCTTG. Glyceraldehyde 3-phosphate dehydrogenase (GAPDH) was used as internal control. For relative quantification, the copy ratios of NM23-H1/GAPDH, VEGF165/GAPDH and MMP-9/GAPDH were calculated and shown as indication of relative expression levels.

### Western blot and immunohistochemistry

Western blot and immunohistochemical staining were performed as described previously (Xu *et al*, 2001) and following the manufacturer's protocols (BD Biosciences), respectively.

### XTT cytotoxicity assay

The cytotoxic effect of berberine on 5-8F, 6-10B and siNM23-H1-transfected 5-8F cells was detected using proliferation kit (XTT II; Boehringer, Mannheim, Germany). Briefly, cells were plated in 96-well culture plates at a density of 5 × 10^4^ cells per well and allowed to attach for 2 h. Berberine was added at various final concentrations (0.35, 0.7, 1.4, 2.8, 5.6, 11.2 and 22.4 *μ*g ml^−1^) in quadruplicate. After 24, 36, 72 and 96 h, 100 *μ*l of XTT reaction solution was added (Iizuka *et al*, 2000). Optical density was read at a wavelength of 490 nm in ELISA plate reader 4 h later.

### Cell migration and invasion assays

The invasion and migration activity of 5-8F, 6-10B and siNM23-H1-transfected 5-8F cells was assayed using a transwell cell culture chamber as described before (Albini *et al*, 1987). Cells were added to transwell chamber (pore size, 12.0 *μ*m; CorningNY, Corning, NY, USA). The number of cells that migrated through the membrane was counted 72 h later. For transfection experiments, cells were seeded 24 h after transfection. For the invasion assays, matrigel (BD Biosciences) was added to the transwell chambers and incubated for 5 h before cells were seeded. The number of membrane-penetrating cells was counted 72 h later. The migration and the invasion assays were performed in quadruplicate for each cell line tested.

### Electrophysiological measurements

Patch-clamp experiments were performed in tight-seal whole-cell configuration at room temperature in a standard bath solution (Wu *et al*, 1998; Li *et al*, 2000). Patch-clamp experiments were recorded with a computer-controlled patch clamp amplifier (Axon). Capacitance and series resistance were calculated with the software-supported internal routines of the Pclamp 8.1 and compensated before each experiment. The voltage-depending calcium electrical current (*I*_Ca_) voltage clamp scheme was square wave, with 30 and −90 mV of voltage, 200 ms of wave width, 10 s of stimulus interval, −90 to +30 mV of command potential and 20 mV of step level pulse. The Ca^2+^-releasing activated Ca^2+^ current (*I*_CRAC_) voltage clamp scheme was square wave, with 0 mV of potential, 200 ms of wave width, 20 s of stimulus interval, −120 to +60 mV of command potential and 20 mV of step level pulse.

### Antimetastasis effect of berberine on aggressive NPC in nude mouse model

Forty nude mice were randomly divided into four groups as shown in Table 4. 5-8F or 6-10B cells were injected subcutaneously into nude mice to produce tumours. Around 1 × 10^7^ tumour cells were injected per animal. Volumes of tumours were measured using a slide calliper, with the volume calculated by the following formula (Hazama *et al*, 1999): *a* × *b*^2^/2, where ‘*a*’ is the larger and ‘*b*’ is the smaller of the two dimensions.

Groups 1 and 2 were injected via tail vein with saline, while groups 3 and 4 were injected with berberine (200 mg kg^−1^ day^−1^) (Xin *et al*, 2005; Shu *et al*, 2006). Nude mice were treated for 7 days before the injection with 5-8F and 6-10B cells. Animal weight and tumour growth were monitored once a week. Four weeks later, mice were killed for implanting tumour tissues preparations. The volume of implanted tumours and body weights of mice were measured to evaluate whether cachexia existed or not.

### Statistical analysis methods

The significance of differences among different measuring indicators was determined with the Student's *t*-test (for normally distributed data), or the Mann–Whitney *U*-test (for nonnormally distributed data). *P*<0.05 was considered as significance. Statistics for the gene chip data was performed by Spotfire 8.0.

## RESULTS

### Microarray analysis of the differentially expressed genes related with NPC intracranial invasion potential in the primary focus tissues

**Figure 1:**
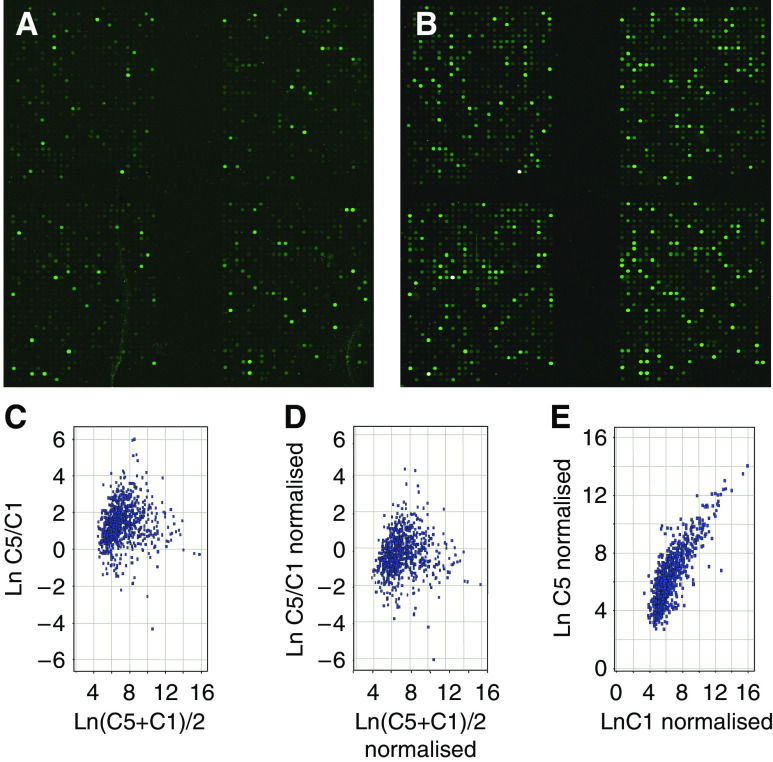
Analysis of the differentially expressed genes associated with intracranial invasion in primary NPC. Note: C5-NPC group with intracranial invasion; C1-NPC group without invasion. (**A**) No invading NPC tissues hybridised atlas of Cy3 and cRNA Xpro HC-III plus labelled; (**B**) Intracranial invading NPC tissues hybridised atlas of Cy3 and cRNA Xpro HC-III plus labelled; the gene-expressing profiling was done three times for each group, and six chips have been performed for the 20 patients. (**C** and **D**) Data normalised scatter plot from the NPC tissue samples with intracranial invading and those with no invasion other than the primary focus. Systemic difference caused by the process of lab was decreased by normalising the compared data. (**E**) Analysing of hybridisation scatter plot of differentially expressive genes in the NPC tissue samples with intracranial invading tendency and those without invasion outside the primary focus. In the panels C, D and E showing the natural logarithm of data from two compared groups as X-axis and Y-axis, difference of two compared groups was judged directly by distribution tendency. The genes distributed along 45° diagonal have been equally expressed, and for those, the vertically further to diagonal, the greater difference was shown in their differential expression.

Gene-expressing profiles of NPC tissues with or without intracranial invasion have been performed three times (Figure 1). There were 31 differentially expressed genes obtained with 8 elevated (overexpressed equal to or more than 2-fold) and 23 downregulated (underexpressed equal or more than 0.5-fold, 50% of control) in NPC tissues with intracranial invasion compared with those without invasion (Tables 2 and 3).

### Validation of differentially expressed genes

**Figure 2:**
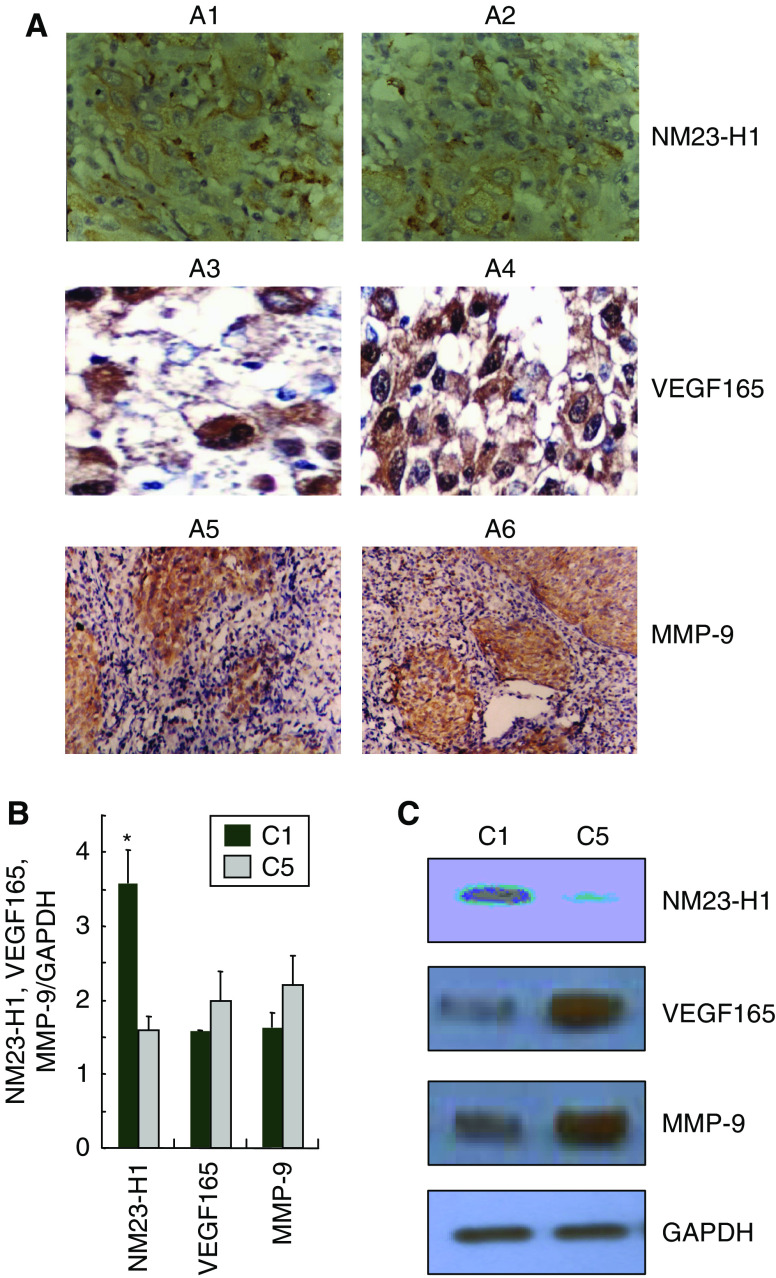
Validation of NM23-H1, VEGF165 and MMP-9 expression in the tissue samples of primary focus of NPC. (**A**) Validation with IHC (H&E, × 10). A1, A3, A5: NPC tissues with no invasion; A2, A4, A6: NPC tissues with intracranial invading signs. (**B**) Validation by the quantitative real-time PCR. Glyceraldehyde 3-phosphate dehydrogenase served as control. Relative mRNA levels of NM23-H1, VEGF165 or MMP-9 mRNA/GAPDH are expressed as the relative abundance. C1: NPC tissues with no invasion, C5: NPC tissues with intracranial invading signs. (**C**) Validation with western blot. Tissues lysates from two groups were subjected to western immunoblotting with anti-NM23-H1, VEGF165 or MMP-9 antibody, and blot was reprobed with anti-GAPDH to verify equal loading.

From the above 31 differentially expressed genes, we chose three genes that is likely to be associated with intracranial invasion of NPC, including NM23-H1, MMP-9 and VEGF165 for validation. NM23-H1 has been shown as a tumour metastasis suppressor in gastric carcinoma, ovarian cancer and other cancers (Kantor *et al*, 1993; Freije *et al*, 1997; Fan *et al*, 2003). MMP-9 has been demonstrated to play a key role in the invading process of cancerous cells (Hofmann *et al*, 2005). Elevated VEGF165 expression might be closely associated with the metastatic potentiality of malignant cells (Cianchi *et al*, 2001; Su *et al*, 2006). To confirm our microarray findings, we used immunohistochemistry (IHC), quantitative real-time PCR and western blot to determine the expression of NM23-H1, MMP-9 and VEGF165 in NPC tissues with and without intracranial invasion. As shown in Figure 2, both mRNA and protein levels of NM23-H1 are significantly lower in NPC tissues with intracranial invasion compared with that without invasion. Meanwhile, we found that both mRNA and protein levels of MMP-9 and VEGF165 are apparent higher in NPC tissues with intracranial invasion (Figure 2).

### Berberine inhibits NPC cell viability and migration

**Figure 3:**
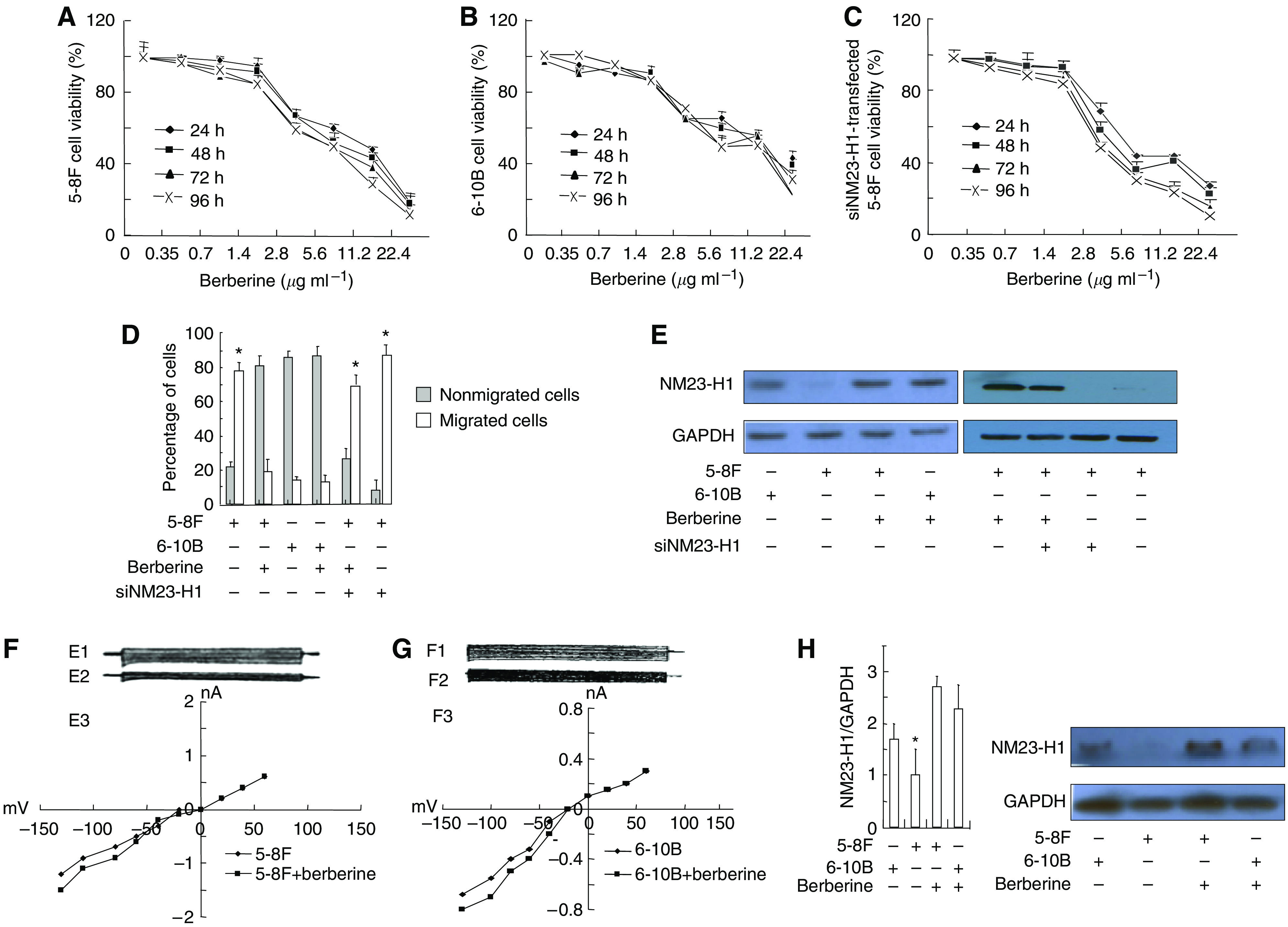
The inhibitory effect of berberine on the NPC invasion *in vitro* and *in vivo*. Cytotoxic effects of berberine on 5-8F (**A**), 6-10B (**B**) or siNM23-H1 transfected 5-8F cells (**C**). The cytotoxicity of berberine was evaluated using an XTT cell proliferation assay. 5 × 10^4^ cells were cultured for 24, 48, 72 and 96 h in 96-well microplates (100 *μ*l per well) with various concentration of berberine as indicated. Cell viability values (%) are expressed as means±s.e. from four separate measurements, *t-*test. (**D**) Inhibitory effect of berberine on cell motility in 5-8F, 6-10B and siNM23-H1 transfected 5-8F cells (cultured with 2.8 *μ*g ml^−1^ berberine for 72 h). The values (% of cells) are expressed as means±s.e. from four separate measurements, *t-*test, ^*^P<0.01, 5-8F, berberine-treated siNM23-H1-transfected 5-8F cells *vs* other cells. (**E**) Berberine increased NM23-H1protein expression in NPC cells, and siNM23-H1 is effective in knocking down the expression of endogenesis NM23-H1. (**F**, **G**) Berberine inhibited *I*_CRAC_ in NPC cells. Igor Pro 4.0 demo was used to observe and record the effect of prolonging time on *I*_CRAC_. (**H**) Left panel: Berberine increased NM23-H1 expression *in vivo*. NM23-H1 mRNA in the implanting tumour tissues of nude mice was shown as the relative abundance and GAPDH as control. Right panel: Berberine increased NM23-H1 protein expression i*n vivo.* Berberine (200 mg kg^−1^ day^−1^) was injected into different groups of nude mice with implanting tumour induced by injection of various lines of NPC cells. NM23-H1 expression level was significantly higher in the tumour tissues from animals induced with 5-8F than those with 6-10B cells.

Berberine is a protoberberine alkaloid, which exists in *Hydrastis canadensis* (golden seal), *Phellodendron amurense*, *Coptis chinensis*, *Berberis vulgaris* (barberry), *Berberis aquifolium* (Oregon grape) and *Berberis aristata* (tree turmeric) (Ikram, 1975). Berberine has been indicated to have inhibitory effect on NPC cells (Iizuka *et al*, 2000; Chan *et al*, 2005). We compared the effect of berberine on cell viability via XTT in two different NPC cell lines, an aggressive one, 5-8F and another one with lower metastasis potential, 6-10B. Time- and dose-dependent cytotoxic effect of berberine on the two cell lines is shown in Figures 3A and B. The viability of berberine-treated 5-8F cells was significantly lower than that of berberine-treated 6-10B cells. We further investigated the effect of berberine on cell motility in 6-10B and 5-8F cells by transwell chamber. As shown in Figure 3D, the number of cells invaded through filters was significantly higher in 5-8F cells than that in 6-10B cells (78±4 *vs* 19±5%, *P*<0.01). When cultured with berberine, the migration of 5-8F cells decreased significantly, but there was no apparent change for 6-10B cells, suggesting that berberine is more potent in suppressing the motility of NPC with high metastasis potential than those with low metastasis potential.

We then examined the expression of NM23-H1 in 6-10B and 5-8F cell lines and found that the NM23-H1 level is much lower in 5-8F cell line. It is plausible that NM23-H1 plays a role in the high metastasis potential of 5-8F cells, and berberine may affect NPC metastasis via NM23-H1. To test this hypothesis, we created several hairpin loop constructs that produce siRNAs targeted against NM23-H1 (siNM23-H1) transcripts and examined the effect of berberine on the cell viability of 5-8F cells transfected with those constructs and compared with that of 5-8F and 6-10B cells. Knocking down of NM23-H1 expression with siRNA significantly increases the viability and migration of berberine-treated 5-8F cells (Figures 3A–D).

Our results suggest that berberine is more effective on aggressive NPC cell line 5-8F, which has low level of NM23-H1. In addition, our data indicate that the anti-NPC effect of berberine is likely to depend on NM23-H1.

### Berberine increases NM23-H1 activity in cells

To confirm our above observation that the anti-NPC effect of berberine depends on NM23-H1, we evaluated the effect of berberine on NM23-H1 expression in NPC cell lines by western blot. As shown in Figure 3E, melittin increased NM23-H1 protein level in 5-8F cells. To further verify the specific effect of berberine on NM23-H1, we introduced siNM23-H1 into 5-8F cells and found that siNM23-H1 reversed the inhibitory effect of berberine on cell motility, as well as NM23-H1 activity (Figures 3D–E).

### Berberine inhibits *I*_CRAC_ in cells

Berberine has been indicated to have inhibitory effect on Ca^2+^ current (Wang *et al*, 1997; Wu *et al*, 1998). It is possible that berberine affects NPC via Ca^2+^ current. To prove that, we examined the effect of berberine on *I*_CRAC_ of 5-8F and 6-10B cell lines by patch clamp. As shown in Figures 3F and G, there were no significant changes in the values of *I*_CRAC_ between 5-8F and 6-10B cells. When treated with berberine, the current value of *I*_CRAC_ in 5-8F cells, significantly decreased from (−1.40±0.37) nA to (−1.28±0.34) nA, (*P*<0.05, see Figure 3F); but there was no apparent change for 6-10B cells (Figure 3G).

### Berberine inhibits NPC growth and metastasis *in vivo*

We have shown above that berberine can induce NM23-H1 expression and prevent the migration of HCC cells. We investigated whether it could suppress HCC tumour growth and induce NM23-H1 expression in implanted tumour induced by injecting 5-8F and 6-10B. The effect of berberine *in vivo* on NPC tumour growth and NM23-H1 expression was determined by examining implanting tumour volume and nude mice body weights (Table 4 and Figure 3H). We found that there was a significant decrease in tumour volumes accompanied by an increase in body weights and NM23-H1 expression in berberine-treated implanted tumour induced by 5-8F cells, suggesting an inhibitory effect of berberine on NPC tumour growth and cachexia. In addition, berberine can induce the expression of NM23-H1 *in vivo*.

## DISCUSSION

Intracranial invasion usually lead to poor prognosis in NPC patients. Therefore, it is important to find out the underlying mechanism for intracranial invasion of NPC. In this study, we used Human Xpro HC-plus cancer-related gene chip to screen intracranial invasion-associated genes for NPC. Several genes that are differentially expressed between NPC with and without intracranial invasion are identified and confirmed including NM23-H1, MMP-9 and VEGF165. They can be used as potential intracranial invasion-associated indicators, especially for NM23-H1. We found that NM23-H1 expression levels are evidently lower in NPC tissues with intracranial invasion as well as with an aggressive NPC cell line, 5-8F, compared with NPC tissues without intracranial invasion and a less aggressive NPC cell line, 6-10B. Knocking down of NM23-H1 expression with RNAi significantly inhibits the cell motility of 5-8F cells. All these evidence indicate that NM23-H1 is an important regulator for the intracranial invasion of NPC cells.

Berberine has been found to have anti-NPC effect and inhibits Ca^2+^ current (Wang *et al*, 1997; Wu *et al*, 1998). We demonstrate that berberine is able to suppress cell viability, cell motility and *I*_CRAC_ of aggressive NPC cells more obviously than that of less aggressive ones. Berberine inhibits cell motility accompanied by induction of NM23-H1 expression. It failed to inhibit cell motility in the siNM23-H1-transfected 5-8Fcells. In addition, berberine could inhibit NPC implanting tumour and cachexia *in vivo* in nude mouse model of human NPC. This evidence indicates that berberine-induced NM23-H1 expression is likely to play a crucial role in its inhibition of cell motility.

In summary, our present investigation has demonstrated that downregulated NM23-H1 expression is very likely to be involved in NPC intracranial invasion. Our finding that berberine can inhibit implanting tumour and cachexia *in vivo* through the induction of NM23-H1 expression, indicates that berberine is a potential drug for NPC.

## Figures and Tables

**Table 1:** Distribution of gender and age in NPC patients

			**Average ages**	**Gender**	
**Groups**	**No. of patients**	**Age ranges**	**(*x̄*±*s*)**	**Male**	**Female**
1	10	20–63	37±3	7	3
2	10	20–63	35±5^*^	7	3

NPC=nasopharyngeal carcinoma.

Note: Group 1, NPC patients with intracranial invasion; group 2, NPC patients without invasion.

^*^P>0.05 compared with group 1.

**Table 2:** Underexpressed genes (<0.5-fold) in intracranial invasive NPC

**Names**	**UniGene_IDs**	**Descriptions**
NM23	79.6	Nonmetastasis cell protein expressed in
CDC25C	656	Cell division cycle 25C
HGF	809	Hepatocyte growth factor
IL8RB	846	Interleukin 8 receptor, beta
IGFBP4	1516	Insulin-like growth factor binding protein 4
NPY	1832	Neuropeptide Y
ELN	9295	Elastin
PTEN	10712	Phosphatase and tensin homolog
GRPR	73883	Gastrin-releasing peptide receptor
APC	75081	Adenomatosis polyposis coli
EXT2	75334	Exostoses (multiple) 2
ST5	79265	Suppression of tumo rigenicity 5
CUL2	82919	Cullin 2
CKS2	83758	CDC28 protein kinase 2
PDGFRL	170040	Platelet-derived growth factor receptor-like
NEO1	90408	Neogenin homolog 1
MXI1	118630	MAX interacting protein 1
CAV2	139851	Caveolin 2
CDK9	150423	Cyclin-dependent kinase 9
BAI2	200586	Brain-specific angiogenesis inhibitor 2
RBM5	201675	RNA binding motif protein 5
TNFSF13B	270737	Tumor necrosis factor superfamily, member 13b
TFE3	274184	Transcription factor binding to IGHM enhancer 3

NPC=nasopharyngeal carcinoma.

**Table 3:** Overexpressed genes (>2-fold) in intracranial invasive NPC tissues

**Names**	**UniGene_IDs**	**Descriptions**
IL11	1721	Tousled-like kinase1
PRDM4	21807	Cytochrome P450, subfamily IIIA (niphedipineoxidase), polypeptide 4
MNT	25497	Adenylate cyclase 6
VEGFC	79141	Vascular endothelial growth factor C
PIM2	80205	Cysteine-rich, angiogenic inducer, 61
NAB2	159223	TXK tyrosine kinase
TPD52L2	154718	Tumor protein p53-binding protein
MMP9	2936	Matrix metalloproteinase 9

NPC=nasopharyngeal carcinoma.

**Table 4:** Comparison of the implanting tumour volumes and the body weights among different groups treated with berberine (*x̄*±*s*)

**Groups**	***n***	**Volumes (cm^3^)**	**Body weights (g)**	**Berberine treatment (200 mg kg^−1^ day^−1^)**	**NPC cells**
1	10	0.375±0.04^*^	23.5±0.44	−	5-8F
2	10	0.219±0.01	25.7±0.47	−	6-10B
3	10	0.206±0.03	28.3±2.59^*^	+	5-8F
4	10	0.183±0.03	26.2±2.42	+	6-10B

NPC=nasopharyngeal carcinoma.

^*^P<0.01 compared with other groups.

## References

[bib1] Albini A, Iwamoto Y, Kleinman HK, Martin GR, Aaronson SA, Kozlowski JM, McEwan RN (1987) A rapid *in vitro* assay for quantitating the invasive potential of tumor cells. Cancer Res 47: 3239–3245 2438036

[bib2] Chan CM, Ma BB, Wong SC, Chan AT (2005) Celecoxib induces dose dependent growth inhibition in nasopharyngeal carcinoma cell lines independent of cyclooxygenase-2 expression. Biomed Pharmacother 59: S268–S271 16507390 10.1016/s0753-3322(05)80043-5

[bib3] Cianchi F, Cortesini C, Bechi P, Fantappiè O, Messerini L, Vannacci A, Sardi I, Baroni G, Boddi V, Mazzanti R, Masini E (2001) Up-regulation of cyclooxygenase 2 gene expression correlates with tumor angiogenesis in human colorectal cancer. Gastroenterology 121: 1339–1347 11729113 10.1053/gast.2001.29691

[bib4] Fan Z, Beresford PJ, Oh DY, Zhang D, Lieberman J (2003) Tumor suppressor NM23-H_1_ is a granzyme A-activated DNase during CTL-mediated apoptosis, and the nucleosome assembly protein SET is its inhibitor. Cell 112: 659–672 12628186 10.1016/s0092-8674(03)00150-8

[bib5] Freije JM, Blay P, MacDonald NJ, Manrow RE, Steeg PS (1997) Site directed mutation of NM23-H1. Mutations lacking motility suppressive capacity upon transfection are deficient in histidine-dependent protein phosphotransferase pathways *in vitro*. J Biol Chem 272: 5525–5532 9038158 10.1074/jbc.272.9.5525

[bib6] Hazama S, Noma T, Wang F, Iizuka N, Ogura Y, Yoshimura K, Inoguchi E, Hakozaki M, Hirose K, Suzuki T, Oka M (1999) Tumor cells engineered to secrete interleukin-15 augment anti-tumour immune responses *in vivo*. Br J Cancer 80: 1420–1426 10424745 10.1038/sj.bjc.6690538 PMC2363078

[bib7] Hofmann UB, Houben R, Brocker EB, Becker JC (2005) Role of matrix metalloproteinase in melanoma cell invasion. Biochimie 87: 307–314 15781317 10.1016/j.biochi.2005.01.013

[bib8] Iizuka N, Miyamoto K, Okita K, Tangoku A, Hayashi H, Yosino S, Abe T, Morioka T, Hazama S, Oka M (2000) Inhibitory effect of Coptidis Rhizoma and berberine on the proliferation of human esophageal cancer cell lines. Cancer Lett 148: 19–25 10680588 10.1016/s0304-3835(99)00264-5

[bib9] Ikram M (1975) A review on the chemical and pharmacological aspects of genus *Berberis*. Planta Med 28: 353–358 1208684 10.1055/s-0028-1097869

[bib10] Kantor JD, McCormick B, Steeg PS, Zetter BR (1993) Inhibition of cell motility after NM23 transfection of human and murine tumor cells. Cancer Res 53: 1971–1973 8481897

[bib11] Lacombe ML (1993) Nucleoside diphosphate kinase/NM_23_ and metastatic potency. Bull Cancer 80: 717–722 8204954

[bib12] Lascu I, Gonin P (2000) The catalytic mechanism of nucleoside diphosphate kinases. J Bioenerg Biomembr 32: 237–246 11768307 10.1023/a:1005532912212

[bib13] Leone A, Flatow U, King CR, Sandeen MA, Margulies IM, Liotta LA, Steeg PS (1991) Reduced tumor incidence, metastasis potential, and cytokine responsiveness of NM23-transfected melanoma cells. Cell 65: 25–35 2013093 10.1016/0092-8674(91)90404-m

[bib14] Li J, Spence KT, Dargis PG, Christian EP (2000) Properties of Ca(2+) release-activated Ca(2+) channel block by 5-nitro-2-(3-phenylpropylamino)-benzoic acid in Jurkat cells. Eur J Pharmacol 394: 171–179 10771282 10.1016/s0014-2999(00)00144-8

[bib15] Ma J, Zhou J, Fan S, Wang L, Li X, Yan Q, Zhou M, Liu H, Zhang Q, Zhou H, Gan K, Li Z, Peng C, Xiong W, Tan C, Shen S, Yang J, Li J, Li G (2005) Role of a novel EGF-like domain-containing gene NGX6 in cell adhesion modulation in nasopharyngeal carcinoma cells. Carcinogenesis 26: 281–291 15498789 10.1093/carcin/bgh312

[bib16] Ng SH, Chang TC, Ko SF, Yen PS, Wan YL, Tang LM, Tsai MH (1997) Nasopharyngeal carcinoma, MRI and CT assessment. Neuroradiology 39: 741–746 9351114 10.1007/s002340050499

[bib17] Nie X, Zhang B, Li X, Xiang J, Xiao B, Ma J, Zhou M, Zhu S, Lu H, Gui R, Shen S, Li G (2003) Cloning, expression, and mutation analysis of NOR1, a novel human gene down-regulated in HNE1 nasopharyngeal carcinoma cell line. J Cancer Res Clin Oncol 129: 410–414 12819961 10.1007/s00432-003-0451-9 PMC12161968

[bib18] Shu D, Wan X, Liu H, Yang J, Zhou Q (2006) Effect of berberine chloride on experimental murine colitis induced by dextran sulfate sodium. J Chin Pharm Sci 15: 182–187

[bib19] Steeg SP, Bevilacqua G, Kopper L, Thorgeirsson UP, Talmadge JE, Liotta LA, Sobel ME (1988a) Evidence for a novel gene associated with low tumor metastatic potential. J Natl Cancer Inst 80: 200–204 3346912 10.1093/jnci/80.3.200

[bib20] Steeg PS, Bevilacqua G, Pozzatti R, Liotta LA, Sobel ME (1988b) Altered expression of NM23, a gene associated with low tumor metastatic potential, during adenovirus 2 Ela inhibition of experimental metastasis. Cancer Res 48: 6550–6554 2460224

[bib21] Su J, Yang P, Yuan J, Yang C, Wei L, Hsieh C, Chou C, Jeng Y, Wang M, Chang K, Hung M, Kuo M (2006) The VEGF-C/Flt-4 axis promotes invasion and metastasis of cancer cells. Cancer Cell 9: 209–923 16530705 10.1016/j.ccr.2006.02.018

[bib22] Sung F, Hui EP, Tao Q, Li H, Tsui NB, Dennis Lo YM, Ma BB, To KF, Harris AL, Chan AT (2007) Genome-wide expression analysis using microarray identified complex signaling pathways modulated by hypoxia in nasopharyngeal carcinoma. Cancer Lett 253: 74–88 17320280 10.1016/j.canlet.2007.01.012

[bib23] Wang J, Zhao L, Lin D (1997) Differentiation-inducing effect of stepholidine and retinoic acid on human head and neck carcinoma. Zhonghua Er Bi Yan Hou Ke Za Zhi 32: 99–102 10743138

[bib24] Workman P, Twentyman P, Balkwill F, Balmain A, Chaplin D, Double J, Embleton J, Newell D, Raymond R, Stables J, Stephens T, Wallace J (1998) United Kingdom Co-ordinating Committee on Cancer Research (UKCCCR) guidelines for the welfare of animals in experimental neoplasia (second edition). Br J Cancer 77: 1–10 10.1038/bjc.1998.1 PMC2151254 9459138

[bib25] Wu S, Yu H, Jan C, Li H, Yu C (1998) Inhibitory effects of berberine on voltage- and calcium-activated potassium currents in human myeloma cells. Life Sci 62: 2283–2294 9651117 10.1016/s0024-3205(98)00209-4

[bib26] Xin H, Wu X, Li Q, Yu A, Zhu M, Zhang Q, Zhu M, Liu Y (2005) Effects of berberine hydrochloride and its coadministration with cyclosporin A on CYP3A2 expression in rat liver and small intestine. Chin Pharm J 40: 353–356

[bib27] Xu Z, Maroney AC, Dobrzanski P, Kukekov NV, Greene LA (2001) The MLK family mediates c-Jun N-terminal kinase activation in neuronal apoptosis. Mol Cell Biol 21: 4713–4724 11416147 10.1128/MCB.21.14.4713-4724.2001 PMC87148

[bib28] Yao K, Shida S, Selvakumaran M, Zimmerman R, Simon E, Schick J, Hass NB, Balke M, Ross H, Johnson SW, O’Dwyer PJ (2005) Macrophage migration inhibitory factor is a determinant of hypoxia-induced apoptosis in colon cancer cell lines. Clin Cancer Res 11: 7264–7272 16243796 10.1158/1078-0432.CCR-05-0135

[bib29] Yih LH, Peck K, Lee TC (2002) Changes in gene expression profiles of human fibroblasts in response to sodium arsenite treatment. Carcinogenesis 23: 867–876 12016162 10.1093/carcin/23.5.867

[bib30] Zeng Z, Zhou Y, Xiong W, Luo X, Zhang W, Li X, Fan S, Cao L, Tang K, Wu M, Li G (2007) Analysis of gene expression identifies candidate molecular markers in nasopharyngeal carcinoma using microdissection and cDNA microarray. J Cancer Res Clin Oncol 133: 71–81 16941191 10.1007/s00432-006-0136-2 PMC12160845

[bib31] Zhao J, Li X (2004) Effects of Fuzheng Yiliu Granule on expression of CD44v6 and nm23-H1 in esophageal carcinoma treated with radiotherapy. J Chin Integr Med 2: 262–264 10.3736/jcim20040406 15339409

